# Automated Bowel Polyp Detection Based on Actively Controlled Capsule Endoscopy: Feasibility Study

**DOI:** 10.3390/diagnostics11101878

**Published:** 2021-10-12

**Authors:** Manh Cuong Hoang, Kim Tien Nguyen, Jayoung Kim, Jong-Oh Park, Chang-Sei Kim

**Affiliations:** 1 School of Mechanical Engineering, Chonnam National University, Gwangju 61186, Korea; hmcuong.hust@gmail.com; 2 Korea Institute of Medical Microrobotics, Gwangju 61011, Korea; kimtiennguyen@chonnam.ac.kr (K.T.N.); jaya@kimiro.re.kr (J.K.); jop@kimiro.re.kr (J.-O.P.)

**Keywords:** active locomotion capsule endoscope, magnetic capsule endoscope, capsule localization, polyp detection, deep learning

## Abstract

This paper presents an active locomotion capsule endoscope system with 5D position sensing and real-time automated polyp detection for small-bowel and colon applications. An electromagnetic actuation system (EMA) consisting of stationary electromagnets is utilized to remotely control a magnetic capsule endoscope with multi-degree-of-freedom locomotion. For position sensing, an electronic system using a magnetic sensor array is built to track the position and orientation of the magnetic capsule during movement. The system is integrated with a deep learning model, named YOLOv3, which can automatically identify colorectal polyps in real-time with an average precision of 85%. The feasibility of the proposed method concerning active locomotion and localization is validated and demonstrated through in vitro experiments in a phantom duodenum. This study provides a high-potential solution for automatic diagnostics of the bowel and colon using an active locomotion capsule endoscope, which can be applied for a clinical site in the future.

## 1. Introduction

The small bowel is the largest digestive organ in the human body and constitutes three-quarters of the length and comprises over 90% of the absorptive surface of the gastrointestinal (GI) tract [1]. However, the diagnosis of cancer in the small bowel is very uncommon in practice, with an overall incidence of 2.3 per 100,000 population in 2019 [2] and 0.6% of all new cancer cases in 2021 [3]. Small-bowel tumors have over 40 different histological types, in which adenocarcinoma has been the most dominant histology of all time, followed by the carcinoid, the lymphoma, and the sarcoma [4]. These tumors arise in all three sections of the small bowel with a 60% distribution rate in the duodenum, followed by the jejunum with 25% to 29% and 10% to 13% in the ileum [2]. The difficulty of small-bowel tumor detection and lack of screening programs cause later-stage diagnoses of these tumors in comparison to that of colorectal cancer (CRC). Based on the investigation of the SEER-Medicare database, 33.7% of patients with small-bowel tumors were diagnosed at the early stage (stage I–II), while the early detection rate of CRC was 52.3% [5]. It is necessary to develop a reliable diagnostic and screening methodology for the small bowel.

A swallowable capsule endoscope (CE) equipped with a digital camera has exhibited its promising potential in GI tract examination since its first clearance at the FDA for clinical usage in 2000 [6]. A wealth of proof has confirmed the contribution of the CE in the increment of diagnosis yield (DY), such as more than 2 million instances of CE utilization and thousands of publications worldwide [7]. The CE has been used to study small-bowel cancer with a reported maximum detection frequency of tumors of 9.5% [8,9]. Recent technological advancement allows the CE to shift from being passively propelled by intestinal peristalsis to actively locomotive or wirelessly manipulated using an external magnetic field control [10,11,12]. These capsules are not only able to perform 3D locomotion but also can collect tissue samples [13,14,15,16,17], marking the target tissue regions (tattooing) [18,19] and facilitating targeted drug delivery [20,21,22,23,24]. 

In addition to the active locomotion ability, the position of the CE during operation gives important information for further post-interventions after abnormality detection by capsule as well as additional functioning. Since the capsule itself cannot provide the position information, an additional tracking system is required for localization. Two main localization methods have been implemented to feedback the CE’s position: the magnetic field strength–based method (using Hall sensor array) [25,26,27] and the electromagnetic-wave-based method (RF coils) [28,29,30,31,32]. 

Based on the locomotion and localization instrumentation, automated diagnostic procedures can be achieved through the CE camera’s image processing. Recently, computer-based diagnosis systems have been studied to improve the DY of the CE. These systems are built up from the supervised machine learning and deep learning algorithms that have been trained on the dataset image samples taken from the CE camera with some gold-standard features of normal and abnormal objects such as color, texture, or shape [33,34]. These systems have demonstrated promising results with high sensitivity and specificity for obscure gastrointestinal bleeding (OGIB) [35,36,37], Crohn disease lesions [38,39], polyps [40,41,42], and so on. Especially for colorectal polyp detection, deep learning models show remarkable performances as summarized in [43]. However, due to the lack of CE motion controllability and position information, tracking the target during diagnostic procedures and marking suspicious regions for post-procedure have not been addressed yet. Nevertheless, the ongoing research on CE technology has demonstrated the promising potential of the CE as a reliable screening tool for small-bowel and colon diagnosis. 

On these backgrounds, the major scientific questions investigated in this work are as follows: (1) what level of technology is required to allow a non-expert operator to navigate a CE in an extreme environment such as the colon or small intestine, and (2) what is the feasibility of an integrated system including all state-of-the-art tools for a full diagnosis of the lower GI tract, which results in reliable data that can be used for further interventions.

**Figure 1:**
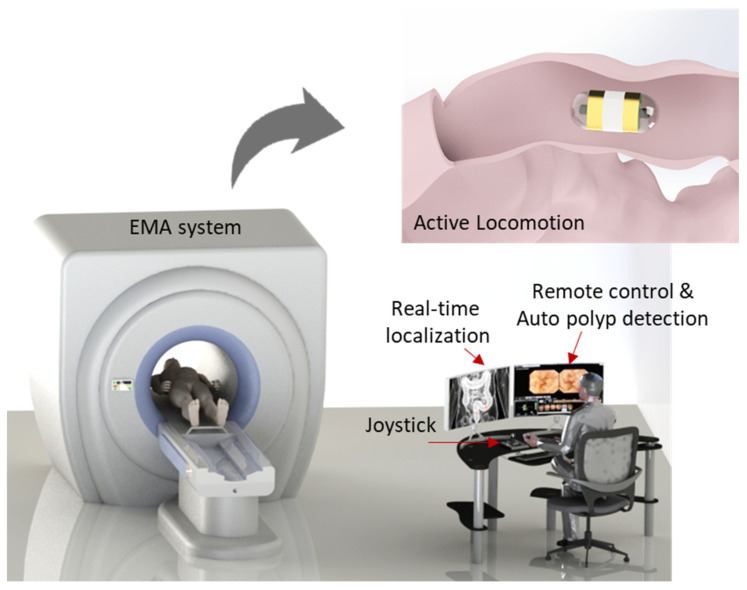
Concept drawing of the overall system.

To successfully cope with these questions, an active locomotion capsule endoscope system including real-time navigation system with position recognition and real-time computer-based polyp detection is designed for the complete diagnosis procedure of the human small bowel and colon (see Figure 1). This could improve the DY of the CE with detailed information regarding disease types and position for further interventions. A control methodology is developed that allows non-specialists to control the magnetic CE with ease based on the CE’s images and CE position feedback from the magnetic sensor array. The proposed methodology is tested in a human-sized small-bowel phantom model setting without an expert user. By doing so, we provide the following contributions: (1) the demonstration of a novel control methodology that enables a non-expert user to control the CE with ease while maintaining performance of the CE comparable to that of experts, and (2) the development of a real-time system for detecting small-bowel and colon polyps with additional information of their location in the GI tract rather than just sensitivity/specificity, which can save time and cost in later interventions.

The proposed approach, being capable of manipulation and navigation, will reduce the dependency of the DY on manual expertise. In addition to being used for the small bowel and colon, this approach can be applied to other endoscopic applications where the environment is known to be a challenge such as the stomach.

## 2. Materials and Methods

### 2.1. Active Control through Electromagnetic Actuation

A magnetic capsule endoscope can be actively controlled by an external magnetic field. In this work, the external magnetic field was created by a multiple electromagnet system known as an electromagnetic actuation (EMA). The configuration of electromagnets was designed to generate a strong magnetic field in the region of interest (ROI) of the system so that the capsule could be controlled in five degrees of freedom (DOFs). Based on the Helmholtz and Maxwell coil configuration, we built the system with a barrel shape so that the patient could easily access it. Figure 1 shows the conceptual design of the system, and a detailed configuration can be found in our previous development [44]. To guarantee a powerful system, we used 10 electromagnets including a pair of Helmholtz coils, 2 pairs Maxwell coils, and 2 pairs of rectangle coils.

Fundamentally, the capsule’s direction is steered by a magnetic torque, τ**,** and the propulsion is caused by a magnetic force, F, as in the following:

(1) τ=VM×B

(2) F=V(M·∇)B 

where B is the external magnetic field and V and M are the volume and magnetization value of the permanent magnet in the capsule. The magnetic torque created by the magnetic field tends to synchronize the direction of the capsule with the magnetic field direction and the magnetic force caused by the magnetic field gradient tends to push it along the heading direction. Based on the demanded alignment torque and propulsion force, the current at the electromagnets can be computed as follows:

(3) $$I={\left[\begin{array}{c} B\left(P\right)\\ M^{T}\partial B_{x}\left(P\right)\\ M^{T}\partial B_{y}\left(P\right)\\ M^{T}\partial B_{z}\left(P\right) \end{array}\right]}^{+}{\left[\begin{array}{c} B\\ F \end{array}\right]}_{desired}=X{\left(P\right)}^{+}{\left[\begin{array}{c} B\\ F \end{array}\right]}_{desired}$$

where $X{\left(P\right)}^{+}$ is the pseudoinverse of the actuation matrix X(P) and B(P), $\partial B_{x}\left(P\right)$, $\partial B_{y}\left(P\right)$, and $\partial B_{z}\left(P\right)$ are unit-current magnetic field and directional gradient field matrices obtained from a simulation model, respectively. These matrices represent the characteristics of the EMA system, including the directional field strength and controllability.

**Figure 2:**
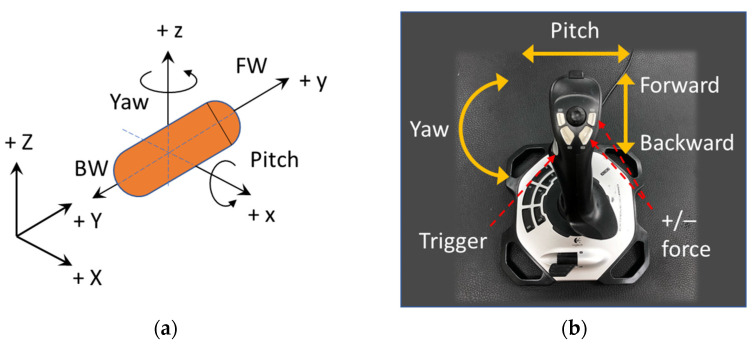
(**a**) Definition of controlled parameters of capsule endoscope and (**b**) mapped joystick functions. FW and BW stand for forward and backward, respectively.

In our previous study [44], ${\left[\begin{array}{cc} B & F \end{array}\right]}^{T}{}_{desired}$ was defined by six parameters. As an advancement from the previous study, we newly present an end-user-oriented control algorithm in this paper. A friendly user interface with fewer control parameters is highly desired, and a simple manipulation methodology should be more intuitive for non-expert users so that the training phase can be shortened. The alignment and propulsion directions of the CE were defined by two variables: pitching angle and yawing angle as shown in Figure 2a. These variables were mapped to functions of a joystick as shown in Figure 2b. After deciding the heading direction, the moving command (forward or backward) was executed by pressing the trigger button on the joystick. For stable movement, the operator must give appropriate values of magnetic field and force, because an inappropriate pair can lead to low control accuracy, especially when the CE is near the boundary of the ROI. Thus, as a result of the behavior examination of the EMA system, we experimentally explored that a ratio of magnetic field B (in Tesla) and gradient field (in Tesla per meter) of 1:10 resulted in a high uniformity of the magnetic field in the ROI and precise control. Therefore, we set γ=1:10 as the default ratio of the magnetic field and gradient field magnitude, and three parameters were left to be controlled by the user, including yaw (y), pitch (p), and pushing force (F). The desired parameters can be described as follows:

(4) $${\left[\begin{array}{c} B\\ F \end{array}\right]}_{desired}=C\left(y,p,F\right)=\left[\begin{array}{c} \gamma Fcos\left(p\right)cos\left(y\right)\\ \gamma Fcos\left(p\right)sin\left(y\right)\\ \begin{array}{c} \gamma Fsin\left(p\right)\\ Fcos\left(p\right)cos\left(y\right)\\ \begin{array}{c} Fcos\left(p\right)sin\left(y\right)\\ Fsin\left(p\right) \end{array} \end{array} \end{array}\right]$$

### 2.2. Position Sensing Based on Hall Effect Sensor Array and Magnetic Field Decomposition

To sense the CE’s position, we used a 2D array of Hall-effect sensors that measured the magnetic strength of a permanent magnet inside the CE. Without the external magnetic field, the position of the CE could be easily obtained using the Hall-sensor-measured data directly for computation. However, under the magnetic actuation condition, the magnetic field at the magnetic sensors was a mixture of the magnet’s and the EMA system’s magnetic field that required a decomposition step to obtain the pure magnetic field of the magnet. 

**Figure 3:**
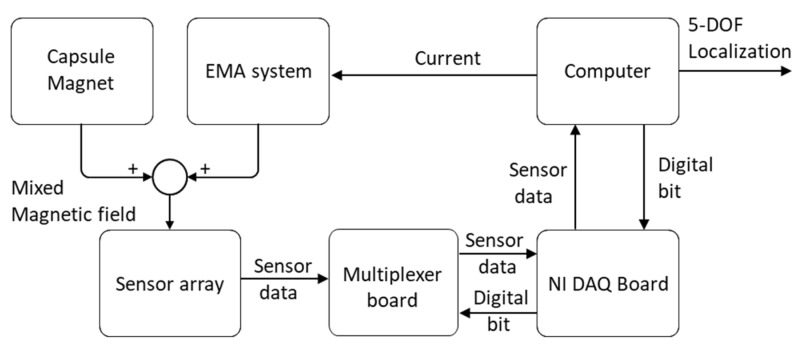
Diagram of data acquisition and compensation method.

Figure 3 shows the diagram of data acquisition of the developed localization system. Inside the ROI of the EMA system, the measured magnetic field of the sensor $H_{S}$ can be described as follows:

(5) $$H_{S}=H_{M}+H_{EMA}$$

where $H_{M}$ is the magnetic field of the capsule’s magnet and $H_{EMA}$ is the external source induced by the EMA system.

The EMA system consisted of 10 electromagnets. Magnetic fields generated by the EMA system were superimposed by the magnetic field of the individual coil and can be computed as the following:

(6) $$H_{EMA}=\overset{10}{\underset{k=1}{\sum }}H_{k}\times i_{k}$$

where $H_{k}$ is the magnetic field of a single electromagnet and $i_{k}$ is the applied current. Clearly, if we know the $H_{EMA}$, we can extract the pure magnetic field of the magnet using the following equation:

(7) $$H_{M}=H_{S}-\overset{10}{\underset{k=1}{\sum }}H_{k}\times i_{k}$$

To do that, the developed system measured the magnetic field of each electromagnet at the unit current. This information was then built into a database that was utilized to reconstruct the magnetic field produced by the EMA system at a given input current from the computer. Different from other research, this developed algorithm does not require information about the specific geometry of the electromagnet and the relative position of the sensor array. This reduces the systematic and measurement errors due to sensor position and electromagnet modeling.

**Figure 4:**
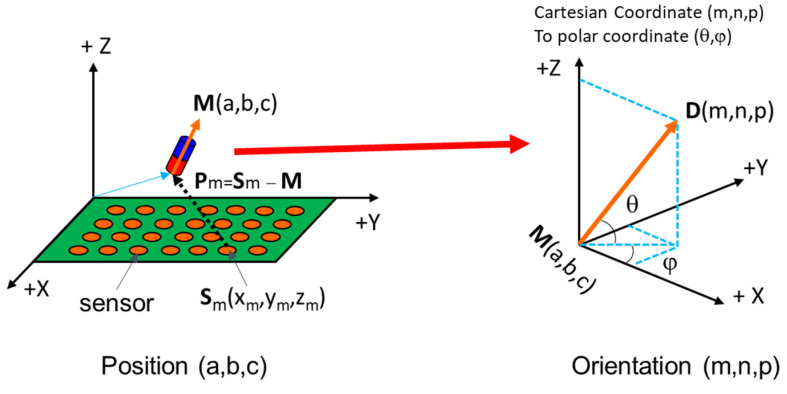
Schematic of a localization system with defined position and orientation of capsule’s magnet.

After removing the external magnetic field of the EMA system, six parameters of position and orientation were computed using an optimization algorithm. We used mono-axis magnetic sensors to measure the magnetic field along the *z*-axis. The magnetic field along the *z*-axis obtained by a sensor at a given position (as shown in Figure 4) was computed as the following:

(8) $$B_{mz}=B_{T}\left(\frac{3\left(D.P_{m}\right)}{\left||P_{m}|\right|{}^{5}}-\frac{D_{z}}{\left||P_{m}|\right|{}^{3}}\right)$$

where ***D***(*m,n,p*) was the magnet’s orientation, $P_{m}=S_{m}\;-\;M$, ***M***(*a,b,c*) was magnet’s position, and $S_{m}$(*x_m_*,*y_m_*,*z_m_*) was the sensor position.

Therefore, the errors along the measured axis were used to establish a cost function as follows:

(9) $$E_{z}=\overset{N}{\underset{m=1}{\sum }}\left(B_{mz}-B_{T}\left(\frac{3\left(D.P_{m}\right)}{R_{m}{}^{5}}-\frac{D_{z}}{R_{m}{}^{3}}\right)\right)$$

The problem can be formulated as finding (*a*, *b*, *c*) and (*m*, *n, p*) such that the square of error is minimized. To solve that nonlinear least-square problem, we applied the Levenberg–Marquardt Algorithm (LMA). The LMA is a trust-region-based optimization method that uses the steepest descent method for global convergence and Newton’s method (quadratic method) for local convergence in a way that gives a smooth transition between the two. The objective function is defined as:

(10) $$\left(a,b,c,m,n,p\right)=\underset{\left(a,b,c,m,n,p\right)}{\arg\;\min}{\left(E_{z}\right)}^{2}$$

The optimization algorithm returns the position *a*, *b*, *c*, and orientation *m*, *n*, *p* of the capsule. The yaw and pitch angles can be obtained from *m*, *n*, *p*.

### 2.3. Deep-Learning-Based Automated Intestinal Polyp Detection

**Figure 5:**
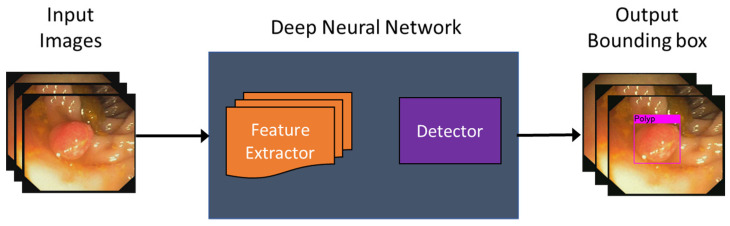
Automated polyp detection diagram using deep neural network. Pink box depicts the detected polyp that is the output of the automated detection model.

We designed a deep-learning-based automated polyp detection software using a public model. The challenge in this work lay in processing the real-time capsule video with a frame rate of 15 to 30 fps, which necessitated a model with high processing time. Furthermore, the neural network needed to be sensitive enough to detect small objects since there are many small polyps at the early stage of the disease. To validate the performance of the proposed method with an active locomotion capsule endoscope with real-time automated polyp detection, we applied a deep neural network model rather than building it from scratch. In this study, we utilized YOLOv3 as a processing library, because it has a good performance in both speed and accuracy [45]. Therefore, YOLOv3 was applied and adapted to detect colorectal polyp in other works [43,46,47]. Basically, the deep neural network feeds the input images through feature extractor blocks followed by a detector; if a polyp was detected, a bounding box surrounding it was overlaid on the input as shown in Figure 5.

YOLOv3 is built on a Darknet53 backbone network used to extract features. For the task of detection, 53 more layers were stacked on top of it, resulting in a 106-layer convolutional network. To deal with various sizes of objects, the YOLOv3 architecture was developed with three prediction heads at three different scales. The detection was done by applying 1 × 1 detection kernels on feature maps of three different sizes at three different places in the network.

Firstly, YOLOv3 divided the image into a 13 × 13 grid and predicted a certain number of bounding boxes with confidence scores corresponding to each grid cell. Then, it used features from the input image to predict the confidence scores for each bounding box. The network predicted several bounding boxes at each grid cell, which may contain polyp, and five parameters of each bounding box including four coordinates of the box around the detected object and their confidence scores. If the bounding box overlapped the ground truth, the prediction score was one and it was lower than 1 when it was less overlapped.

## 3. Results

### 3.1. Experimental Setup

**Figure 6:**
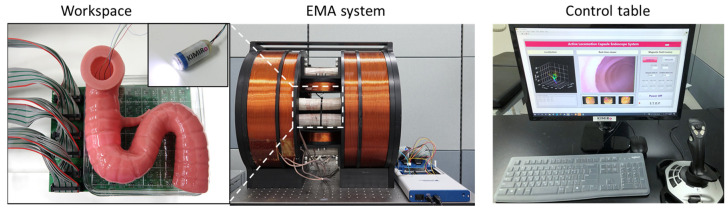
Photoshoot of the real experiment setting.

Figure 6 shows the experimental setting. Ten electromagnets were connected to ten power supplies (including six MX15 and four 3001iX units from AMETEK, Berwyn, PA, USA) that were controlled by a central computer through the GPIB communication protocol. The user interface with the control algorithm was programmed in LabVIEW 2017. A small camera was mounted inside the EMA system to record the movements of the capsule from the top view.

The capsule prototype for this experiment had a dimension of 30 mm (L) × 12 mm (D). An N54 NdFeB magnet with an outer diameter and length of 10 and 12 mm, respectively, was used. The magnet was fixed inside the capsule body so that the magnetic moment of the prototype was along capsule’s body axis. We used a wired camera inside the capsule prototype, the wire was to power the camera and transmit the images to the computer. The wired camera was connected to the computer via an image acquisition device. The image resolution was 720 × 480 pixels, at a frame rate of 30 fps.

The magnetic sensor array consisted of 6 × 6 linear mono-axis Hall-effect sensors (DRV5055, Texas Instruments, Dallas, TX, USA), which cover 100 mm × 100 mm under the ROI of the EMA system. The sensor had a low noise output, a sensitivity of 12.5 mV/mT, and a measurement range of ±169 mT. Due to the limited number of analog channels of the DAQ device (NI USB-6351, National Instruments, Austin, TX, USA), a six-multiplexer board (74H4051, Texas Instruments) was designed to collect the sensor data and feed it to the computer through the DAQ. The test phantom was fixed above the sensor board. All motions and controlled parameters were given through the joystick. A manual control strategy was performed in all experiments.

### 3.2. Active Locomotion with Position Sensing

**Figure 7:**
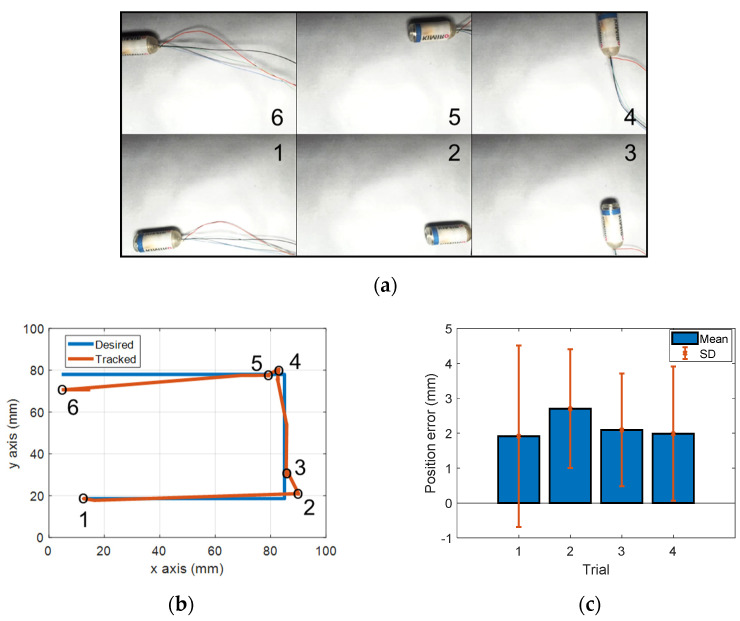
Demonstration of active locomotion in free space. (**a**) Snapshot of capsule robot’s position, (**b**) tracking and desired position of capsule, (**c**) statistical analysis of the movement accuracy with mean and standard deviation (SD) in four trials. The numbers 1, 2, 3, 4, 5, 6 represent the positions of capsule endoscope.

First, we investigated the active locomotion performance of the system. To demonstrate and statistically evaluate the movements of the capsule controlled remotely by the EMA system, the capsule was manipulated in free space as shown in Figure 7a. The capsule was put in an open acrylic box that was 10 mm above the sensor board. A magnetic field of 5 mT was applied to align the capsule endoscope along the moving direction, and a gradient field of 50 mT/m was used for propelling it along the planned path. The capsule was remotely controlled to move along the U-shape trajectory. The experiment was repeated four times. Figure 7a demonstrates the results of active locomotion of the first trial with specific locations of the capsule robot in the desired path. Motion from 1 to 2 was backward (moving direction was opposite to heading direction of capsule), motions 3–4 and 5–6 were forward. Figure 7b shows the desired path and tracking result of the first trial. The results in Figure 7a,b show that the controlled trajectory of the capsule was highly matched with the desired path. The statistical analysis of movement error of four trials is illustrated in Figure 7c. In the first trial, the mean error was 1.91 mm, and the SD was 2.59 mm. The maximum mean error and SD were 2.7 mm (of the second trial) and 2.59 mm (of the first trial), respectively. These results show a high control accuracy and repeatability of the developed system. Position error occurred in some segments of the path, for example, point 5–6. Several reasons caused the control errors, including disturbances, stiffness of wire, uncontrollable rolling motion of capsule, and high distortion of the magnetic field at the edge of ROI. However, compared to the large ROI, these errors were insignificant and acceptable.

**Figure 8:**
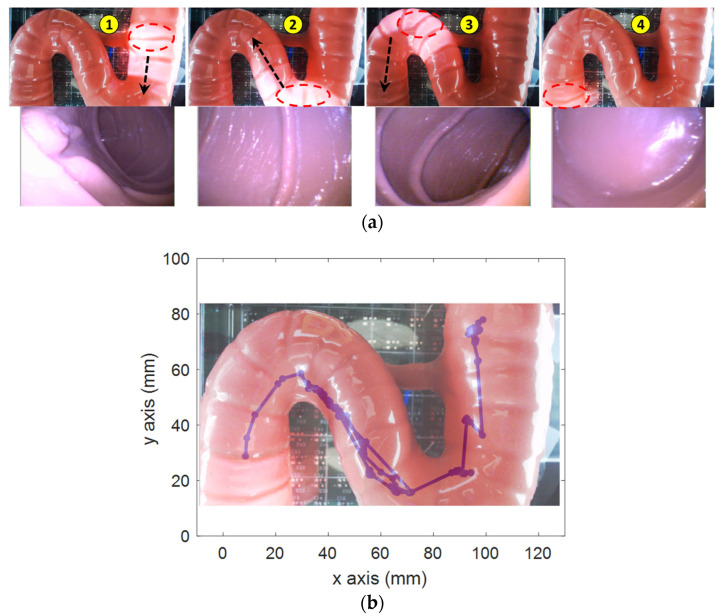
Demonstration of active locomotion in a small-bowel phantom. (**a**) Position of the CE inside the phantom where the light from capsule camera (red dash line) exposes its current position and the extracted pill’s camera images corresponding to positions 1, 2, 3, and 4. The black arrow shows the moving direction. (**b**) Overlaid phantom photo and localization data. The purple dot and the curve represents the tracked position.

Second, the active locomotion test was conducted in a small-bowel phantom (Duodenum piece, EsophagoGastroDuodenoscopy simulator LM-103) as shown in Figure 8. The phantom was fixed on top of the sensor array. The operator used the controls to move the capsule to the end of the phantom based on the pill camera. Due to the friction force between the capsule and the phantom, a higher magnetic field and gradient field of 7 mT and 70 mT/m, respectively, were used. The operator firstly steered the capsule direction so that the lumen was at the center of the image; then, they pushed it along the heading direction. Figure 8a demonstrates the representative positions and corresponding captured images from the CE’s camera. The operator successfully moved the capsule through the high-curvature sections and scanned the entire duodenum with capsule robot vision. Figure 8b shows the localization data, whose shape was qualitatively similar to that of the phantom. 

**Figure 9:**
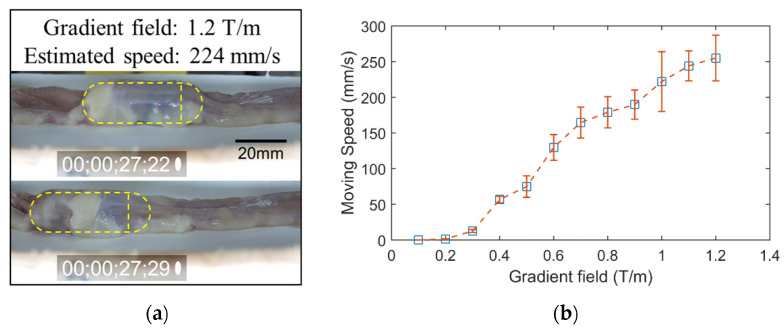
(**a**) Snapshots of capsule movement in the small bowel; the timer is of the format hour; min; sec; frame. The yellow dash line shows the position of capsule endoscope. (**b**) Estimated moving speed of the CE in pig’s small bowel.

Third, we investigated the locomotion performance of the proposed system in a segment of fresh porcine small bowel (purchased from a local slaughterhouse). To estimate the performance of the CE in the small bowel, which is the most difficult organ to move in due to the narrow shape and high viscosity, we estimated the moving speed with respect to levels of the gradient magnetic field. The CE was put inside a segment of a collapsed pig’s small bowel. The test object was put inside the ROI of the EMA system, and the recording camera captured the motion of the capsule at a rate of 60 fps. The magnetic field was created to align the CE along the *y*-axis, and the gradient magnetic field was increased from 0.1 to 1.2 T/m (maximum gradient field value of the system) with a step of 0.1 T/m. At each level, we repeated the experiment 10 times; the average speed was estimated for every iteration. Figure 9a demonstrates the movements of CE in the small bowel when the input gradient magnetic field value was 1.2 T/m. Figure 9b shows the average speed and standard deviation of levels of the gradient magnetic field. It was confirmed that the EMA system can produce a strong magnetic field and force to propel the CE to move in a collapsed small bowel and that the speed of CE can be controlled by adjusting the gradient magnetic field. The system can reach a remarkable value of 250 mm/s moving speed. This is a significant advantage of the proposed system, the endoscopic diagnosis time in the small bowel can thus be shortened.

**Figure 10:**
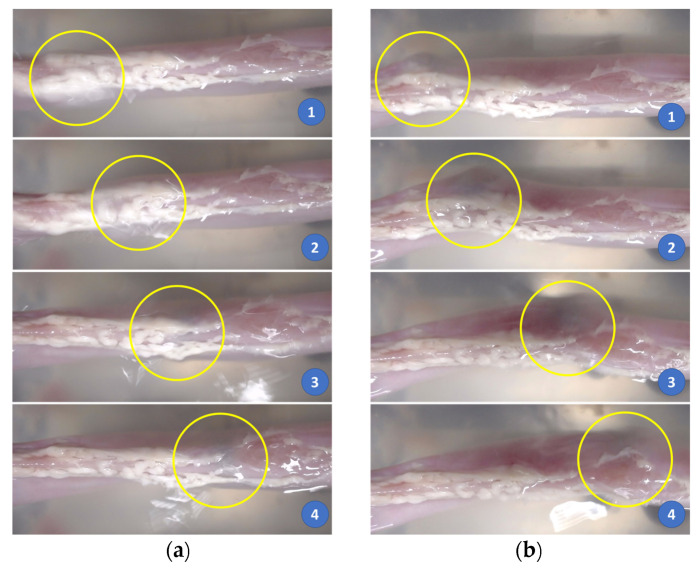
Movement of the CE in the water-submerged small bowel with different magnetic field gradient inputs. (**a**) Gradient field = 0.2 T/m. (**b**) Gradient field = 0.4 T/m. The yellow circle depicts the position of the CE.

Finally, we submerged the small bowel in a water tank and evaluated the locomotion performance of the proposed system and the effect of its motion on the subject. The small bowel was fixed at its two ends to the edges of the tank, while its body was afloat in water. In the previous test, the small bowel was collapsed on a base that resisted the movement of the CE. In this experiment, the small bowel was free when being flooded with water, and we evaluated the effect of the capsule movements on the subject. We aligned the small bowel along the *y*-axis of the system. To test the effect of the magnetic field modulation on the small bowel, we aligned the capsule along the *y*-axis with a magnetic field value of 0.02 T and pushed it at different values of the gradient magnetic field. Figure 10 demonstrates the motions of the capsule robot with different command inputs. With a 0.2 T/m gradient magnetic field (the ratio γ=1:10), we observed that capsule’s motion had an impact on the small bowel, but it was insignificant, the shape of the small bowel was almost unchanged as shown in Figure 10a. However, when the gradient magnetic field was 0.4 T/m (γ=1:20), the capsule’s motion had a noticeable impact on the small bowel as shown in Figure 10b. This was due to the high distortion of the magnetic field when an inadequate ratio *γ* was used, as discussed in Section 2.1. In conclusion, using a good combination of magnetic field and force can guarantee high-accuracy movement, which minimizes the impact on the intestinal organs.

### 3.3. Automated Detection of Intestinal Polyp

To validate the performance of automated polyp detection, we used the CVC-CLINIC database (from Hospital Clinic, Barcelona, Spain) for training and the ETIS-LARIB database for testing [48]. The CVC-CLINIC data were obtained using the Olympus Q160AL and Q165L with Exera II videoprocessor and ETIS-LARIB (Lariboisière hospital, Paris, France) database were obtained by a device Pentax 90i series with EPKi 7000 videoprocessor. The training dataset contained 612 frames having at least one polyp and the testing set contained 199 instances containing a polyp. Images were 384 × 288 pixels with three channels of colors (red, green, blue). The ground truth of each image was labeled by an expert and provided in the data, where the white areas marked polyp’s locations. Firstly, the ground truth images were used to extract the bounding box, which was defined as the input to the YOLOv3 model. The training dataset was small, we used the data augmentation technique with angle, saturation, exposure, and hue feature to increase the number of training datasets. The learning rate was set at 0.001 with 0.9 momenta and 0.005 decay rate.

**Figure 11:**
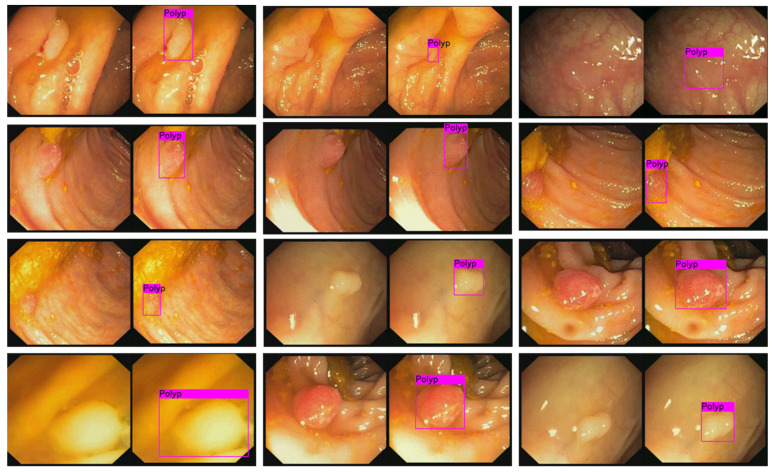
Pairs of original images and detected polyp images. Pink box represents the detected polyp that is the output of the automated detection model.

Figure 11 illustrates the results of detected polyp in images from the tested dataset. It was successful in identifying polyps of different sizes. In addition, to evaluate the goodness of the model, we also computed the average precision (AP) and mean of the intersection of union (IOU). We got 85% in AP and 69.47% in IOU on the tested dataset. With this result, there were also several failure cases of correct polyp detection. This might be due to the small size of the training data and the non-optimized hyper-parameters during the training phase.

By combining the automated polyp detection technique and the localization function as demonstrated above, once the polyp was detected, the software stored the current position of the capsule endoscope with a captured image of the polyp as historical data for the next intervention.

## 4. Discussion and Conclusions

In this study, we addressed a complete methodology capable of autonomous abnormality detection in the small bowel and colon with active locomotion and position recognition. The capsule can be remotely controlled in 5-DOFs, moving through and scanning the lower GI tract by a joystick with an end-user UI with only three control parameters. The computer-aided pill perception can automatedly detect and show the polyps in real time with an acceptable accuracy, and the localization system can precisely locate their positions through capsule position sensing.

The small bowel and colon have the same geometric shape—tubular. The scanning procedure of the small bowel and colon is similar, i.e., by simply moving the MCE for-ward/backward along the centerline of the organs due to the tubular shape and the large field of view of the pill camera (140 degrees). In addition, the colon has a bigger diameter and a more lubricated surface than the small bowel (the friction coefficient of a pig colon and small bowel are in the order of 10^−3^ [49] and 10^−1^ [50,51], respectively). Therefore, the proposed system can be potentially applicable to the colon. From a practical point of view, it is more rigorous to investigate the locomotion in the worst possible circumstances (i.e., high-friction-force environment). For this reason, the small bowel has been used to demonstrate the performance of the system. As shown in Section 3.2, the capsule endoscope can be moved in a collapsed small bowel at a speed up to 250 mm/s and in a water-submerged one precisely. It can, therefore, be implied that the proposed system can be applied in the colon. 

The system is proposed to control capsule endoscopes in the small bowel and colon, which have many folds. When moving through the folding structure, the movement of the CE might push and impact the organs, which may cause some abnormal feelings to the patient. With the aid of the localization system and capsule perception, an advanced control algorithm can be employed to deal with that problem and make the procedure more comfortable. The localization system provides feedback data that can be used to develop an autonomous locomotion capsule endoscope. The system can generate input commands itself using the capsule’s camera and computer vision algorithm. The controller will take input commands and feedback data to compute the currents at the electromagnets.

The proposed system is safe since it can be turned off in case of malfunctions or system failure. Due to the linearity properties of the coil, the magnetic field can be completely removed by shutting down the power supply system. The magnetic field used to control the capsule is significantly lower than that in MRI scanner systems. MRI scanner systems can use 1.5 T or up to 4.7 T [52] magnetic fields for imaging, while the proposed system can control the capsule at 0.02 T magnetic field and 0.2 T/m gradient field. It is not possible to conclude about the adverse effects of magnetic exposure of the developed system on the human body, and the system needs safety tests for clinical application. However, the field strength implies that the impacts are smaller than that of conventional MRI systems in the clinical site. 

There remain some limitations in this study. In the view of clinical application, the proposed technology is still complex and requires a huge system compared to the commercially available digestive organ diagnostic technology. However, the proposed hardware platform can achieve wireless capsule motion control of a capsule that can overcome the passive movement of the conventional capsule, such as a commercialized system (ANKON, China). The proposed automated detection algorithm may enhance the usability and efficacy of the current state of wirelessly controllable capsule endoscopy technology. The following are several advantages of the proposed methodology: (1) controllable magnetic field for safe operation, (2) five degrees of freedom of motion control of a capsule, and (3) shortened diagnostic time by active motion and automated detection, additionally, the proposed method can have a benefit even with a huge size. Moreover, the presented end-user-oriented control for simple and easy operation can provide a more intuitive terminal operation environment to an operator including ones with no expertise. Nevertheless, the system size and complexity need to be revisited further for more clinician-friendly settings.

The localization system can track the CE in the ROI of 100 mm × 100 mm × 40 mm, but when the CE is higher than 40 mm above the sensor board, the tracking accuracy decreases due to the limited sensing range of the sensors. The accuracy of the automated polyp detection was not significantly high; the dataset was not sufficient due to the limited access to the databases. The resultant detection rate of 85% is not the best; however, it is comparable to the results in other works with the APs ranging from 63.8% to 91.64% [45,48]. In this study, we focus on proposing a complete solution for the advanced monitoring of the bowel and colon by integrating an active locomotion capsule endoscope, tracking capsule position, and automated polyp detection. We put relaxed the expectations on the performance of the detection model at the current research stage. The detection rate can be improved in the future by building a better model and using a bigger dataset. The proposed method does not fully demonstrate the integrated performances in vivo; however, essential technical components could be fully validated through in vitro studies and academically available datasets.

In the future, this study will be extended to an autonomous scanning of the lower GI tract, where the capsule robot can give the actuation command itself based on the visual perception and localization data while the detection algorithm will identify the abnormalities with their location. Although we discussed the potential applicability at the colon, it should be validated through experiments. Moreover, the proposed method will be validated in animal experiments for actual clinical application.
